# Intermittent Diabetes Care and the Risk of Diabetic Kidney Disease in Patients with Type 2 Diabetes Mellitus: Findings from Data Analysis of Local Governmental Claims Data

**DOI:** 10.31662/jmaj.2020-0106

**Published:** 2021-07-06

**Authors:** Yuta Takahashi, Yukio Suzuki, Soshi Dohmae, Hiroshi Murayama

**Affiliations:** 1 Health and Welfare Bureau, City of Yokohama, Kanagawa, Japan; 2 Medical Care Bureau, City of Yokohama, Kanagawa, Japan; 3 Department of Obstetrics and Gynecology, Yokohama City University Graduate School of Medicine, Kanagawa, Japan; 4 Research Team for Social Participation and Community Health, Tokyo Metropolitan Institute of Gerontology, Tokyo, Japan

**Keywords:** diabetic kidney disease, intermittent treatment, type 2 diabetes mellitus, claims data

## Introduction

Globally, public health measures have shifted to a new stage, Public Health 3.0 ^(1)^, which emphasizes the concept of community-wide prevention. The active use of accessible data and their application to policies, particularly by local governments, are necessary to achieve this concept.

In Japan, preventing the onset and exacerbation of diabetic kidney disease (DKD), one of the major diseases that necessitates dialysis, is important as the number of patients with diabetes increases ^(2)^. Many previous studies have reported a strong correlation between intermittent diabetes treatment and DKD onset and exacerbation ^(3)^. However, most studies determined the intermittent treatment status through a patient questionnaire survey, which requires both time and money and may be affected by reporting bias, recall bias, and follow-up loss.

This study investigated the association between intermittent diabetes treatment and DKD onset in patients with type 2 diabetes mellitus using highly objective and comprehensive claims data. Herein, we defined intermittent diabetes treatment as the interruption of antidiabetic prescriptions for a certain time period.

## Materials and Methods

### Study design

Data were obtained from the large medical claims database in Yokohama City (Yokohama Original Medical Database; YoMDB) ^(4)^, including the National Health Insurance and Medical Care System for the Elderly aged ≥75 years. As of September 2020, the population of Yokohama City was approximately 3.75 million.

**Figure 1.**
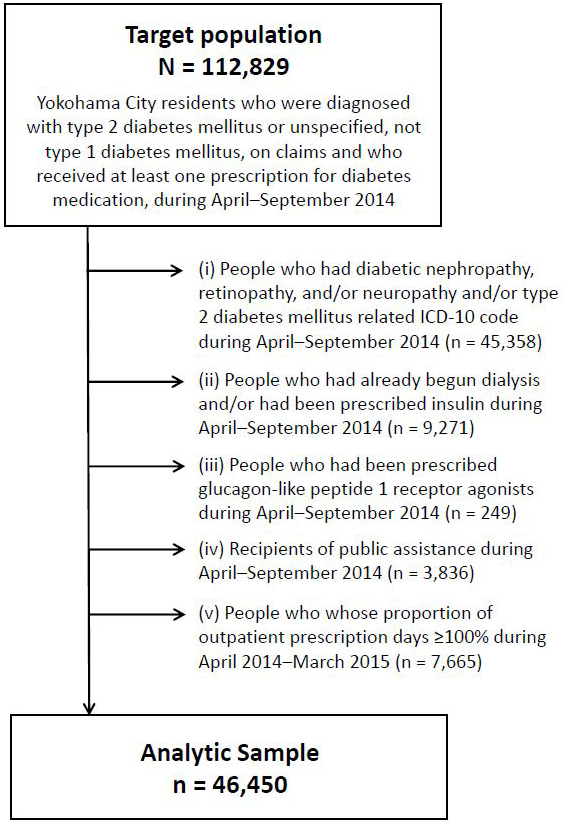
Flow diagram of the selection process of the study participants.

Data of participants who were diagnosed with type 2 diabetes mellitus or unspecified diabetes (ICD-10 codes E11/E14), not type 1 diabetes mellitus, as per claims made during April-September 2014 (hereafter, baseline period) and received at least one prescription for diabetes medication during the baseline period (n = 112,829) were evaluated. We excluded 66,379 participants based on the following criteria: (i) microvascular complications of diabetes, such as diabetic nephropathy, retinopathy, neuropathy, and/or type 2 diabetes mellitus-related ICD-10 codes (E11.0-E11.9) during the baseline period; (ii) initiation of dialysis and/or prescription of insulin during the baseline period (because these factors partly reflect diabetes severity); (iii) prescription of glucagon-like peptide-1 receptor agonists during the baseline period (because there were no robust rules for its prescription during the baseline period in Japan and this might not reflect diabetes severity); (iv) the use of public assistance (*seikatsu-hogo* in Japanese) during the baseline period (because all their medical costs were covered by the local government); and (v) the proportion of outpatient prescription days exceeding 100% during April 2014-March 2015 (details described in the next section). Consequently, 46,450 patients were analyzed. Figure 1 shows the flow diagram of the selection process of the study participants.

The study protocol was approved by the Institutional Ethics Committee of Yokohama City University School of Medicine (B180700010). This study used an opt-out system at the official website of Yokohama City, instead of obtaining informed consent from patients.

### Measures

Intermittent diabetes treatment was calculated as the proportion of days during which diabetes medication (excluding insulin) was prescribed during a 1-year period (April 2014-March 2015). Participants were divided into tertiles according to the proportion: low prescription (≤73.96%), moderate prescription (73.97-92.05%), and high prescription (≥92.06%). The outcome was DKD onset (ICD-10 codes E112 and E142). Participants were followed up for 4 years (April 2014-March 2018), the duration until prescriptions were recorded on claims data was determined. The covariates included sex, age, and diagnoses of hypertension, dyslipidemia, and hyperuricemia, which were identified to be associated with DKD ^(5)^, during the baseline period.

### Statistical analyses

Cox proportional hazards models were used to calculate the relative mortality risk of each intermittent treatment category, setting the “high-prescription” category as the reference. For sensitivity analyses, we additionally ran the models (i) after excluding those with a proportion of outpatient prescription days of <50% and (ii) including those with a proportion of outpatient prescription days of ≥100%. All covariates were controlled in the model. The results are shown as the adjusted hazard ratios (aHRs) with 95% confidence intervals (CIs). The analyses were performed using the EZR version 1.41 ^(6)^.

## Results

**Figure 2.**
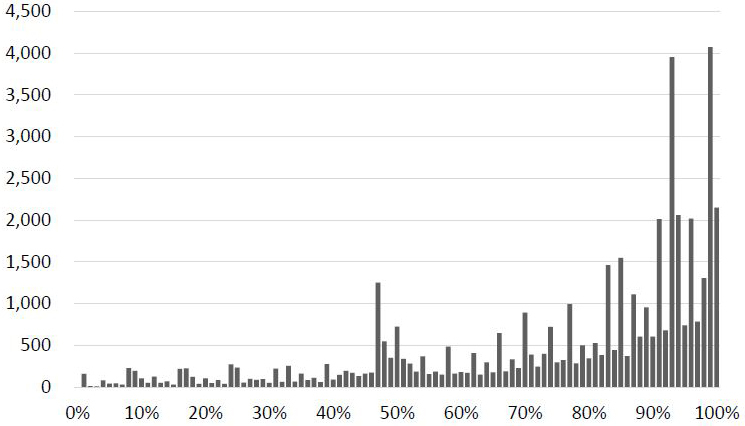
Distribution of the proportion of outpatient prescription days.

Table 1 shows the participants’ background characteristics. Overall, 4.6% of participants were newly diagnosed with DKD during the observation period. Men and those aged ≥80 years accounted for 55.8% and 24.3% of the patients, respectively. The average proportion of outpatient prescription days was 76.2% (standard deviation [SD], 23.9). Figure 2 shows the distribution of the proportion of outpatient prescription days.

**Table 1.** Characteristics of the Study Participants.

	Total sample (n = 46,450; 100.0%)	No new onset of diabetic kidney disease (n = 44,294; 95.4%)	New onset of diabetic kidney disease (n = 2,156; 4.6%)
	n (%)/mean ± SD	n (%)/mean ± SD	n (%)/mean ± SD
Sex			
Men	25,922 (55.8)	24,610 (55.6)	1,312(60.9)
Women	20,528 (44.2)	19,684 (44.4)	844 (39.1)
Age			
≤49 years	1,705 (3.7)	1,632 (3.7)	73 (3.4)
50-59 years	2,561 (5.5)	2,443 (5.5)	118 (5.5)
60-69 years	11,824 (25.8)	11,252 (25.4)	572 (26.5)
70-79 years	19,084 (41.1)	18,195 (41.1)	889 (41.2)
≥80 years	11,276 (24.3)	10,772 (24.3)	504 (23.4)
Hypertension			
Yes	35,056 (75.5)	33,376 (75.4)	1,680 (77.9)
No	11,394 (24.5)	10,918 (24.6)	476 (22.1)
Dyslipidemia			
Yes	32,545 (70.1)	31,006 (70.0)	1,539 (71.4)
No	13,905 (29.9)	13,288 (30.0)	617 (28.6)
Hyperuricemia			
Yes	6,773 (14.6)	6,375 (14.4)	398 (18.5)
No	39,677 (85.4)	37,919 (85.6)	1,758 (81.5)
Proportion of outpatient prescription days (%)	76.2 ± 23.9	76.1 ± 24.0	79.6 ± 20.7
T1 (low prescription: ≤73.96%)	15,431 (33.2)	14,820 (33.5)	611 (28.3)
T2 (moderate prescription: 73.97-92.05%)	13,950 (30.0)	13,219 (29.8)	731 (33.9)
T3 (high prescription: ≥92.06%)	17,069 (36.7)	16,255 (36.7)	814 (37.8)

SD: standard deviation.

Compared to the high-prescription group, the low-prescription group showed a lower risk of DKD after adjusting for covariates (aHR [95% CI]: 0.84 [0.75-0.93]; Table 2). The association between the moderate-prescription group and the outcome was not significant (1.01 [0.92-1.12]). Male sex, hypertension, and hyperuricemia were also risk factors for DKD.

**Table 2.** Adjusted Hazard Ratios of Risk Factors for Diabetic Kidney Disease.

		Total sample		Excluding those with a proportion of outpatient prescription days of <50%	
		aHR	(95% CI)	aHR	(95% CI)
Sex^a^	Men	1.22	(1.12-1.34)	1.19	(1.08-1.31)
Age^b^	50-59 years	1.05	(0.79-1.41)	1.02	(0.73-1.42)
	60-69 years	1.10	(0.86-1.40)	1.07	(0.81-1.42)
	70-79 years	1.08	(0.85-1.37)	1.09	(0.83-1.43)
	≥80 years	1.14	(0.89-1.47)	1.13	(0.85-1.50)
Hypertension^c^	Yes	1.13	(1.02-1.25)	1.12	(1.00-1.25)
Dyslipidemia^d^	Yes	1.02	(0.92-1.12)	1.01	(0.92-1.12)
Hyperuricemia^e^	Yes	1.27	(1.13-1.42)	1.27	(1.13-1.43)
Proportion of outpatient prescription days^f^	T1 (low prescription: ≤73.96%)	0.84	(0.75-0.93)	1.05	(0.93-1.19)
	T2 (moderate prescription: 73.97-92.05%)	1.01	(0.92-1.12)	1.01	(0.92-1.12)

aHR: adjusted hazard ratio; CI: confidence interval. ^a^ Reference: Women ^b^ Reference: Age ≤49 years ^c^ Reference: No hypertension ^d^ Reference: No dyslipidemia ^e^ Reference: No hyperuricemia ^f^ Reference: T3 (high prescription: ≥92.06%)

In the sensitivity analyses, we excluded patients with a proportion of outpatient prescription days of <50% (n = 7,498). The risk estimate in the low-prescription group was attenuated and became insignificant (1.05 [0.93-1.19]; Table 2). Moreover, we included those with a proportion of outpatient prescription days of ≥100% (n = 7,572) in the total analysis sample. The risk of new DKD onset in this group did not differ significantly from that in the high-prescription group (1.06 [0.93-1.20]; data not shown in the table).

## Discussion

This study revealed that people in the low-prescription group had a 16% lower risk of DKD during the 4-year study period than those in the high-prescription group. However, after excluding patients with a proportion of outpatient prescription days of <50%, our study did not identify any significant difference in DKD risk among the low-, moderate-, and high-prescription groups. Patients with a low proportion of outpatient prescription days (<50%) tended to be healthy and had a low risk of new DKD onset. Previous studies have shown that patient adherence would affect complications ^(3), (7)^; thus, this study suggests that the proportion of outpatient prescription days in the local governmental claims data might not be a proxy of adherence.

In Japan, local governments maintain national health insurance and medical claims data and can analyze these data to evaluate their health policies. Studies such as ours can contribute to efficient and evidence-based policymaking.

This study had some limitations. First, DKD onset was identified using only claims data; the actual number of patients with DKD may have been underestimated. In Japan, the diagnosis code in claims data does not necessarily indicate the actual diagnosis by a physician. However, previous studies have reported the validity of diagnosis from the large-scale claims database ^(8), (9), (10)^; we expect that our results did not deviate significantly from the true prevalence and association. Second, the association between intermittent treatment and DKD onset may be overestimated because of an inability to control residual confounders, including diabetes severity, history of hospitalization related to diabetes or diabetic complications, disease duration (time since diagnosis), and uncontrolled glucose levels. Third, given the natural course of progression to DKD, the follow-up period (4 years) might be too short to detect DKD onset ^(11)^.

In conclusion, using local governmental claims data, we found that a lower proportion of outpatient prescription days was associated a lower risk of DKD in patients with type 2 diabetes mellitus; however, this risk was attenuated after excluding those with a proportion of outpatient prescription days of <50%. Our analytical findings were minimally affected by bias because we used highly objective and complete claims data. Future evidence-based policies are likely to prioritize the use of claims data registered by local governments.

## Article Information

### Conflicts of Interest

None

### Acknowledgement

The authors thank all the members of the Health and Welfare Bureau and Medical Care Bureau of the City of Yokohama, Japan, for their cooperation in this study.

### Author Contributions

YT and YS designed the study and wrote the draft of the manuscript. All the authors contributed to the preparation of the manuscript and data analyses. All the authors have critically reviewed the manuscript and approved the final version of the manuscript.

### Approval by Institutional Review Board (IRB)

This study protocol was approved by the Institutional Ethics Committee of Yokohama City University School of Medicine (B180700010).
